# Trends in Opioid Misuse Among Individuals Aged 12 to 21 Years in the US

**DOI:** 10.1001/jamanetworkopen.2023.16276

**Published:** 2023-06-01

**Authors:** Lauren Klein Warren, Joella Adams, Georgiy Bobashev

**Affiliations:** 1 RTI International, Durham, North Carolina

## Abstract

**Importance:**

Although opioid misuse has been decreasing among US youths and adolescents in recent years, it is unclear what has contributed to this trend and how this trend differs by age group and sex over time.

**Objective:**

To identify trends in opioid misuse among youths and young adults across and between ages, birth cohorts, and sexes.

**Design, Setting, and Participants:**

Cross-sectional National Survey on Drug Use and Health (NSDUH) public-use files were used to produce nationally representative pseudocohorts. The survey population includes the civilian US population in the 50 states and Washington, DC. Individuals without a fixed address and institutionalized individuals were excluded. Respondents to the NSDUH are a population-based sample selected using a stratified cluster design. For the years (January 1, 2002, to December 31, 2019) and ages (12-21 years) analyzed, the sample sizes ranged from 1607 to 3239 respondents. Data were analyzed from January 1, 2022, to April 12, 2023, for the main outcome by age, sex, and pseudocohort.

**Main Outcomes and Measures:**

Respondents were asked whether they misused prescription opioids or used heroin in the past year. The analysis hypotheses were formulated and tested after data collection.

**Results:**

In a total of 5 pseudocohorts, data from 114 412 respondents aged 12 to 21 years were analyzed; the unweighted distribution of male sex (complement was female) ranged from 47.7% to 52.6% (mean [SD], 50.6% [1.1%]). Response rates ranged from 45.8% to 71.3%. High school–aged youths and young adults had distinctly lower rates of opioid misuse in later pseudocohorts compared with earlier ones. Rates of misuse among individuals aged 16 years were 2.80% (95% CI, 1.06%-4.54%) higher in 2002 vs 2008; among those aged 18 years, rates were 4.36% (95% CI, 1.85%-6.87%) higher in 2002. Similarly, rates of misuse among individuals aged 16 years were 3.93% (95% CI, 2.15%-5.71%) higher in 2008 vs 2014; among those aged 17 years, rates were 3.41% (95% CI, 1.94%-4.88%) higher in 2008. Similar patterns were observed by sex. In earlier cohorts, younger female participants had higher rates of opioid misuse than their male counterparts and older male participants had higher rates than their female counterparts. Sex differences decreased in later cohorts.

**Conclusions and Results:**

The findings of this cross-sectional study of US youths and young adults suggest that high school–aged individuals consistently misused fewer opioids in later pseudocohorts overall and by sex. Sex differences in opioid rates also diminished in later pseudocohorts. A decrease in drug availability and general exposure to the harms of opioid use could be contributing to these findings. Future planned research using this pseudocohort approach will examine polysubstance use and evaluate how substance use differs by other sociodemographic characteristics.

## Key Points

**Question:**

How have opioid misuse prevalence trajectories changed for youths and young adults by birth cohorts in the US from 2002 to 2019?

**Findings:**

In this cross-sectional study of 114 412 participants, the trajectories of opioid misuse in youths and young adults improved based on cross-sectional pseudocohorts beginning in 2002, 2005, 2008, 2011, and 2014. Most notably, during high school years, later pseudocohorts had lower starting prevalence rates, and the rates increased less sharply with age.

**Meaning:**

These findings describing pseudocohort prevalence rates for opioid misuse, including a decrease in opioid misuse in high school years in recent pseudocohorts, may provide insight into behaviors that affect drug use trends among youths and young adults in the US household population.

## Teaser

This cross-sectional study uses National Survey on Drug Use and Health data to identify trends in opioid misuse among US youths and young adults from 2002 to 2019.

## Introduction

The national epidemic of drug overdose has been devastating and worsening over time, with over 107 000 deaths in 2021.^1^ Treatment for opioid use disorder and many harm reduction measures, while important, do not directly reduce opioid misuse for individuals who do not have an opioid use disorder or who use opioids infrequently. Early exposure to opioid misuse increases the likelihood of future problematic use and a transition to heroin use.^2,3^ However, substance use among youths and adolescents is often associated with experimentation, and a large proportion of those who experiment do not continue to use substances into adulthood.^4,5^ Although opioid misuse has been decreasing among youths and adolescents in recent years,^6,7^ it is unclear what has contributed to this trend and how this trend differs by age group and sex over time.

Studying cohorts can better our understanding of observed trends in opioid use and inform national drug policy about future drug behaviors. Positive cohort trends (eg, reduction in opioid misuse) can be the result of public interventions such as media campaigns or school-based programs and more general awareness of the harms associated with opioid use. Negative cohort trends (eg, earlier age of first opioid misuse) can illuminate emerging patterns in use and/or a change in risk factors. Improved understanding of cohort trends can guide future intervention and public health efforts to decrease the risk of opioid-related harms.

The effect of intervention initiation efforts can be difficult to evaluate at the national level because of multiple, overlapping prevention efforts (eg, youth campaigns, prescription drug monitoring efforts) and exposure to general media coverage of problems related to substance use and, later, the overdose epidemic. Because these efforts have been implemented at different times and to varying degrees locally, differential cohort effects may be present. Although successful prevention strategies exist,^8,9^ current prevention measures may not do enough to avert substance use initiation.^10^ National survey data can provide insights into the potential impact of ecological factors, including prevention messaging, treatment utilization, and mortality trends on substance use initiation and other prevention-related outcomes. In this study, our objective was to identify and better understand the association of cohorts and trends in opioid misuse among adolescents.

## Methods

### Source Data: 2002 to 2019 National Survey on Drug Use and Health

The National Survey on Drug Use and Health (NSDUH) collects substance use and mental health data from the US civilian, noninstitutionalized population every year. In this pseudocohort analysis, we focused on a single age per year from January 1, 2002, to December 31, 2019. Across all year and age combinations for the 5 pseudocohorts generated, the sample sizes for the public-use files ranged from 1607 to 3239 respondents per age and year. Data on race and ethnicity were available on the NSDUH data sets, but analyses using these data were outside the scope of the present research. We focused on past-year opioid misuse per age and year across the pseudocohorts. We adhered to the components listed in the Strengthening the Reporting of Observational Studies in Epidemiology (STROBE) guideline for cross-sectional studies where applicable. Review by an institutional review board was not applicable for our study, because our analyses used publicly available, deidentified data available on the Substance Abuse and Mental Health Services Administration website.

Our primary outcome variable was opioid misuse. This outcome was constructed based on an affirmative response to either having misused prescription pain relievers or using heroin in the past year. These measures have undergone statistical imputation and have no missing data. Among the individuals in our analysis sample who misused opioids in the past year, only 3.1% used heroin in the past year and 0.7% used heroin without also misusing prescription pain relievers. Estimates of prevalence rates and variances were calculated using the survey design variables and analytic weights to obtain nationally representative estimates and accurately estimate uncertainty. Because this was a secondary data analysis on publicly available data, information on other components that are not present in this report can be found on the NSDUH web page.^11^

### Development of Pseudocohorts

In general, repeated cross-sectional studies do not follow up specific individuals over time. However, because of the national-level representativeness of large national surveys and through person-level analysis weights, it is possible to track behaviors of individuals in pseudocohorts. Pseudocohorts are single-age subpopulations. For example, individuals who were 18 years of age in 2003 would have been 19 years of age in 2004. Thus, through generating single-year subpopulations, it is possible to examine long-term trajectories, not of specific individuals but of pseudocohorts in general. Although these 19-year-old individuals were not the same 18-year-old individuals we observed the year before, they were representative of the underlying population of young adults this age. Analyzing these representative samples provides insight into what behaviors we observe in this entire population during these years and how these behaviors change as people increase in age. A similar pseudocohort approach has been used in the past to measure marijuana use among adults^12^ and substance use trends among youths.^13^

Public-use NSDUH data contain individual ages starting from 12 to 21 years of age; after 21 years of age, data are collapsed into age groups. To create pseudocohorts for youths and young adults, we set a starting point at which the individuals in our sample were 12 years of age and categorized them by age and year until (1) the cohort reached age 21 years or (2) we reached the 2019 survey year (ie, the final NSDUH year we analyzed). We thus produced multiple pseudocohorts of individuals defined by the starting year to evaluate how substance use patterns evolved. We evaluated 5 pseudocohorts, which we defined as follows:

- 2002 Pseudocohort, born in 1990, aged 12 years in 2002 and 21 years in 2011;
- 2005 Pseudocohort, born in 1993, aged 12 years in 2005 and 21 years in 2014;
- 2008 Pseudocohort, born in 1996, aged 12 years in 2008 and 21 years in 2017;
- 2011 Pseudocohort, born in 1999, aged 12 years in 2011 and 20 years in 2019 (the final NSDUH year we analyzed); and
- 2014 Pseudocohort, born in 2002, aged 12 years in 2014 and 17 years in 2019 (the final NSDUH year we analyzed).

### Statistical Analysis

Data were analyzed from January 1, 2022, to April 12, 2023. We evaluated opioid misuse behaviors for the 5 pseudocohorts by analyzing the data for each year as the underlying population aged with time. We computed and graphed the percentages of past-year misuse of opioids across time for each relevant age. We also performed and documented results from statistical comparisons of the percentages of opioid misuse among specific years between cohorts. We repeated this analysis for male and female participants separately and compared opioid misuse rates between sexes for each age and pseudocohort.

We used SAS software, version 9.4 (SAS Institute Inc), and SUDAAN software, version 11.0.4 (Research Triangle Institute), to produce design-adjusted 95% CIs and to perform pairwise testing of prevalence rates between cohorts and between sexes. The questions asked in the NSDUH that were used to produce the opioid misuse outcomes are found in eTable 4 in Supplement 1. Two-sided α < .05 indicated statistical significance.

## Results

### Results by Age and Pseudocohort

**Figure 1.**
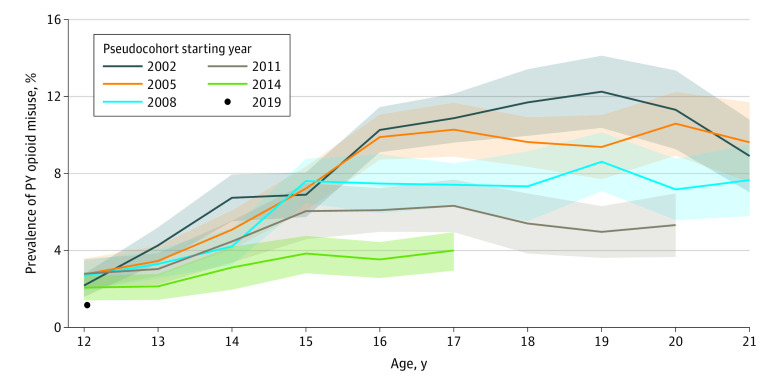
Prevalence of Past-Year (PY) Opioid Misuse by Pseudocohort and Age Shaded areas represent 95% CIs.

Data from 114 412 respondents aged 12 to 21 years were analyzed; the unweighted distribution of male sex for this sample ranged from 47.7% to 52.6% (mean [SD], 50.6% [1.1%]; complement was female sex). Figure 1 presents the prevalence of opioid misuse by age and pseudocohort. Past-year prevalence of opioid misuse gradually increased with age in each pseudocohort before eventually flattening. Table 1 shows that in our earliest pseudocohort—beginning with participants aged 12 years in 2002—the prevalence of past-year opioid misuse was 2.18% (95% CI, 1.59%-2.78%) at 12 years of age and peaked at 19 years of age at 12.25% (95% CI, 10.38%-14.11%). For comparison, in the 2011 pseudocohort, the prevalence of past-year opioid misuse among those aged 12 years was 2.79% (95% CI, 2.04%-3.54%) and increased to only 4.96% (95% CI, 3.63%-6.29%) for those aged 19 years.

**Table 1.** Prevalence of Past-Year Opioid Misuse by Age and Cohort^a^

Age, y	Prevalence, % (95% CI)				
	2002 Cohort	2005 Cohort	2008 Cohort	2011 Cohort	2014 Cohort
12	2.18 (1.59-2.78)	2.76 (1.94-3.57)	2.68 (1.92-3.44)	2.79 (2.04-3.54)	2.07 (1.46-2.68)
13	4.26 (3.32-5.19)	3.46 (2.68-4.24)	3.30 (2.55-4.06)	3.04 (2.23-3.84)	2.13 (1.49-2.77)
14	6.73 (5.52-7.93)	5.08 (4.08-6.07)	4.21 (3.26-5.16)	4.46 (3.39-5.54)	3.12 (2.04-4.19)
15	6.90 (5.74-8.05)	7.22 (6.08-8.35)	7.60 (6.45-8.75)	6.04 (4.59-7.49)	3.84 (2.93-4.75)
16	10.27 (9.11-11.44)	9.89 (8.73-11.05)	7.47 (5.94-9.01)	6.10 (4.98-7.22)	3.54 (2.67-4.42)
17	10.88 (9.62-12.14)	10.28 (8.89-11.68)	7.41 (6.30-8.53)	6.32 (4.96-7.67)	4.00 (3.06-4.94)
18	11.69 (9.97-13.40)	9.63 (8.34-10.92)	7.33 (5.52-9.14)	5.40 (3.84-6.96)	NA
19	12.25 (10.38-14.11)	9.37 (7.72-11.02)	8.61 (7.10-10.13)	4.96 (3.63-6.29)	NA
20	11.31 (9.28-13.34)	10.59 (8.94-12.24)	7.17 (5.59-8.75)	5.32 (3.68-6.95)	NA
21	8.91 (7.03-10.79)	9.62 (7.55-11.68)	7.65 (5.79-9.51)	NA	NA

Abbreviation: NA, not applicable.

^a^ Data are from the 2002-2019 National Survey on Drug Use and Health public-use files. The 2011 cohort included individuals aged 20 years in 2019 and the 2014 cohort included those aged 17 years in 2019, the last year of NSDUH data analyzed.

Figure 1 and statistical testing results in Table 2 and Table 3 show high school–aged youths and young adults (aged 14-18 years) had distinctly lower rates of opioid misuse in later pseudocohorts vs earlier ones. High school–aged individuals in the 2008 pseudocohort had lower rates of opioid misuse than their counterparts in the 2002 cohort. Table 3 shows that the decrease in opioid misuse prevalence between the 2008 and 2002 pseudocohorts was 2.52% (95% CI, 0.97%-4.06%) for those aged 14 years, 2.80% (95% CI, 1.06%-4.54%) for those aged 16 years, 3.47% (95% CI, 1.85%-5.08%) for those aged 17 years, and 4.36% (95% CI, 1.85%-6.87%) for those aged 18 years. Similarly, high school–aged individuals in the 2014 pseudocohort had lower rates of past-year opioid misuse compared with their counterparts in the 2008 pseudocohort. Table 3 shows the decrease in opioid misuse prevalence between the 2014 and 2008 pseudocohorts was 3.76% (95% CI, 2.28%-5.23%) for those aged 15 years, 3.93% (95% CI, 2.15%-5.71%) for those aged 16 years, and 3.41% (95% CI, 1.94%-4.88%) for those aged 17 years. We did not make comparisons between the 2014 and 2008 pseudocohorts for individuals older than 17 years because 17 years of age corresponds to year 2019, which was the latest year we analyzed.

**Table 2.** Comparison of Past-Year Opioid Misuse Prevalence Between Adjacent Cohorts by Age^a^

Age. y	2005 vs 2002 Cohorts		2008 vs 2005 Cohorts		2011 vs 2008 Cohorts		2014 vs 2011 Cohorts	
	Difference, % (95% CI)	*P* value	Difference, % (95% CI)	*P* value	Difference, % (95% CI)	*P* value	Difference, % (95% CI)	*P* value
12	−0.57 (−1.59 to 0.44)	.26	0.08 (−0.80 to 0.95)	.86	−0.11 (−1.24 to 1.02)	.84	0.73 (−0.24 to 1.70)	.14
13	0.79 (−0.43 to 2.02)	.20	0.16 (−0.92 to 1.24)	.77	0.27 (−0.88 to 1.41)	.65	0.91 (−0.13 to 1.94)	.08
14	1.65 (0.08 to 3.22)	.04^b^	0.87 (−0.46 to 2.20)	.20	−0.25 (−1.55 to 1.04)	.70	1.35 (−0.18 to 2.88)	.08
15	−0.32 (−1.93 to 1.29)	.70	−0.38 (−1.79 to 1.03)	.60	1.56 (−0.30 to 3.42)	.10	2.20 (0.45 to 3.94)	.01^b^
16	0.38 (−1.29 to 2.06)	.65	2.41 (0.24 to 4.59)	.03^b^	1.37 (−0.53 to 3.28)	.16	2.56 (1.19 to 3.92)	<.001^b^
17	0.60 (−1.39 to 2.58)	.55	2.87 (1.10 to 4.63)	.001^b^	1.10 (−0.67 to 2.86)	.22	2.31 (0.62 to 4.01)	.007^b^
18	2.05 (−0.11 to 4.22)	.06	2.31 (0.07 to 4.54)	.04^b^	1.92 (−0.52 to 4.36)	.12	NA	NA
19	2.88 (0.29 to 5.47)	.03^b^	0.75 (−1.50 to 3.00)	.51	3.65 (1.55 to 5.76)	.001^b^	NA	NA
20	0.72 (−1.79 to 3.23)	.57	3.42 (1.12 to 5.71)	.003^b^	1.85 (−0.66 to 4.37)	.15	NA	NA
21	−0.70 (−3.51 to 2.11)	.62	1.97 (−0.53 to 4.46)	.12	NA	NA	NA	NA

Abbreviation: NA, not applicable.

^a^ Data are from the 2002-2019 National Survey on Drug Use and Health public-use files. The 2011 cohort included individuals aged 20 years in 2019 and the 2014 cohort included those aged 17 years in 2019, the last year of NSDUH data analyzed. *P* values have not been adjusted for multiple comparisons.

^b^ Statistically significant at α < .05.

**Table 3.** Comparison of Past-Year Opioid Misuse Prevalence Between Selected Cohorts by Age^a^

Age, y	2002 vs 2008 Cohorts		2008 vs 2014 Cohorts	
	Difference, % (95% CI)	*P* value	Difference, % (95% CI)	*P* value
12	−0.49 (−1.47 to 0.48)	.32	0.61 (−0.37 to 1.59)	.22
13	0.95 (−0.25 to 2.16)	.12	1.17 (0.18 to 2.17)	.02^b^
14	2.52 (0.97 to 4.06)	.001^b^	1.09 (−0.35 to 2.53)	.13
15	−0.70 (−2.41 to 1.01)	.42	3.76 (2.28 to 5.23)	<.001^b^
16	2.80 (1.06 to 4.54)	.002^b^	3.93 (2.15 to 5.71)	<.001^b^
17	3.47 (1.85 to 5.08)	<.001^b^	3.41 (1.94 to 4.88)	<.001^b^
18	4.36 (1.85 to 6.87)	.001^b^	NA	NA
19	3.63 (1.22 to 6.05)	.003^b^	NA	NA
20	4.13 (1.55 to 6.72)	.002^b^	NA	NA
21	1.27 (−1.39 to 3.92)	.35	NA	NA

Abbreviation: NA, not applicable.

^a^ Data are from the 2002-2019 National Survey on Drug Use and Health public-use files. We did not make comparisons between the 2014 and 2008 pseudocohorts for individuals older than 17 years because 17 years of age corresponds to year 2019, which was the latest year we analyzed. *P* values have not been adjusted for multiple comparisons.

^b^ Statistically significant at α < .05.

The percentages of past-year opioid misuse were most similar across cohorts at 12 years of age (the range of prevalence rates at this age across the 5 pseudocohorts we analyze was 2.07%-2.79%), but the separation between cohorts increased with age, peaking at 19 years of age. The range of past-year opioid misuse rates among pseudocohorts was 2.13% to 4.26% at 13 years of age, 3.84% to 7.60% at 15 years of age, 4.00% to 10.88% at 17 years of age, and 4.96% to 12.25% at 19 years of age. This demonstrates that the differences between pseudocohorts grow as adolescents become older.

### Results by Sex

**Figure 2.**
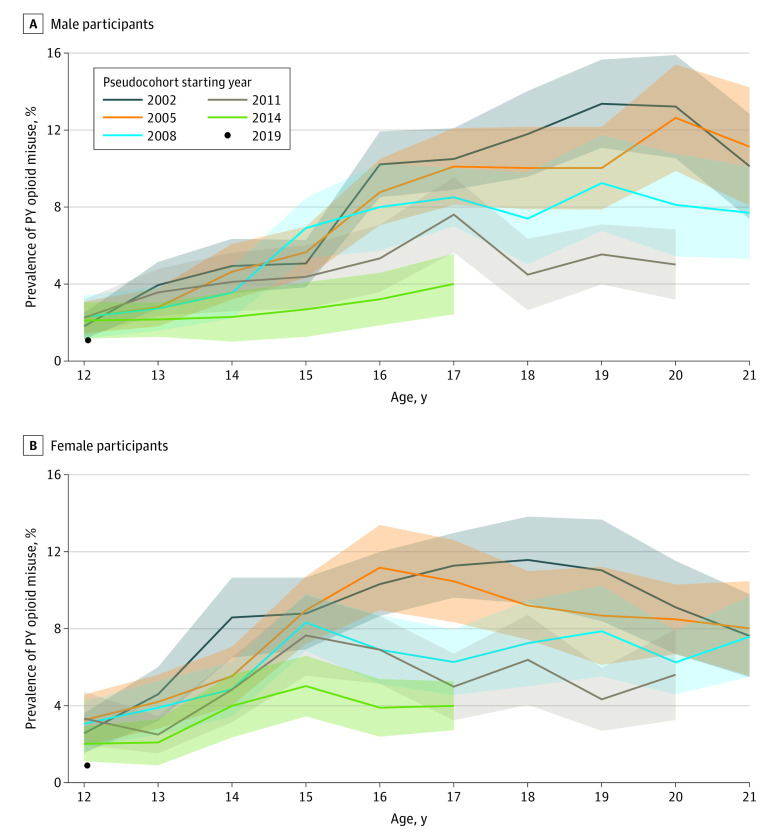
Prevalence of Past-Year (PY) Opioid Misuse by Sex and Age Shaded areas represent 95% CIs.

The same pattern of decreased opioid misuse for later pseudocohorts was observed for high school–aged male and female individuals separately. Additionally, Figure 2A and B and eTables 1 and 2 in Supplement 1 show that female individuals tended to have a peak in opioid misuse at an earlier age than their male counterparts, but male individuals sustained higher rates of use than female counterparts in later ages.

Sex differences that were observed in earlier pseudocohorts did not persist in later cohorts, when the prevalence rates decreased for both sexes (eTable 3 in Supplement 1). In the earlier pseudocohorts (ie, pseudocohorts beginning in 2002 and 2005), male individuals had significantly lower rates of past-year opioid misuse than female counterparts at 15 years of age (difference in rates, −3.73% [95% CI, −5.83% to −1.62%] in the 2002 cohort and −3.30% [95% CI, −5.36% to −1.23%] in the 2005 cohort). In the 2002 pseudocohort, we observed the differences move to the opposite direction as the youths got older. Past-year opioid misuse rates in this pseudocohort were 4.11% higher for male individuals than among female counterparts at 20 years of age (95% CI, 1.04%-7.19%). In later cohorts, the differences between sexes are not as distinct, in part because the overall rates were lower in later cohorts.

## Discussion

Using a pseudocohort approach, we calculated population-level opioid misuse trajectories that indicate a change in opioid misuse among youths and young adults over the last 2 decades. Our main finding is that opioid misuse in adolescence has decreased over time both with respect to overall rates and peak opioid misuse rate. This finding holds for both male and female youths and young adults.

This reduction in opioid use over time could have multiple explanations. One possible explanation is that there was a shift toward an older mean age of initiation for the later cohorts. However, we found no shift in the mean age of first use of opioids. Another potential explanation could be related to the increase in treatment (ie, greater access to treatment and increased treatment success could be responsible for the reduction in past-year use). Analysis of lifetime treatment utilization^14^ showed that the percentage of youths and young adults who received substance use treatment in their lifetime across ages decreased slightly in later pseudocohorts. Because access to treatment did not increase, this would not explain the trends of decreased opioid misuse among the pseudocohorts. We also checked exposure to prevention messages^2^ and again observed that although the exposure remained high (>80%), exposure to prevention messaging decreased in later cohorts.

One possible contributing factor to the opioid misuse reductions we observed is that the perceived ease of obtaining heroin (according to the youths or young adults who were surveyed) decreased across the pseudocohorts (ie, youths found it more difficult to obtain heroin and also misused fewer opioids in more recent pseudocohorts). eTable 4 in Supplement 1 displays the NSDUH questions used in this analysis.

We found that the gap in past-year opioid misuse among male and female participants narrowed in later cohorts, even while overall substance use decreased. This narrowing of sex differences over time has been observed in other nationally representative surveys. The Monitoring the Future Panel Study annual report found that sex differences have narrowed over time for marijuana use, alcohol use, and binge drinking.^15^ Findings from the National Longitudinal Alcohol Epidemiologic Survey suggest that differences between the sexes regarding the hazard of alcohol use and dependence are decreasing in more recent cohorts.^16^ Theories to explain this narrowing have focused on lower gender role traditionality (eg, more women in the workforce, increased access to contraception) and increasingly similar access to substances over time.^17^

Our overall finding of reduced opioid misuse in later pseudocohorts may follow a larger trend of decreasing sexual behavior and risky substance use in US adolescents over time. A recent report summarizing trends observed within the Youth Risk Behavior Survey of the Centers for Disease Control and Prevention from 2009 to 2019 reported declines in the percentage of students who ever had sex, had 4 or more sex partners, and ever used or injected drugs.^18^ Rates of teenage pregnancy,^19^ juvenile arrests,^20^ and hazardous automobile driving^21^ have all decreased compared with previous years. Although it is unclear what is driving this overall trend, hypotheses include improved parent-child relationships, increased use of electronic media, and a concomitant decrease in unstructured socializing.^21^ Notably, some patterns are trending in the opposite direction, including negative mental health and expressed concerns for safety.^18^ In summary, although it should be reassuring that some trends related to substance use are trending positively, there is need to recognize the changing pressures that youths and adolescents face related to mental health and anxiety, which may pose future challenges related to substance use.

Although other studies have reported a decrease in misuse of opioids among adolescents over time,^6,7^ this analysis incorporates population growth curves and adds to the evidence by tracking the timing of use, peak age of use, and periods of decreased use, which may signal experimental or short-term use. We also quantified the shift in use by age and explored risk and protective factors for potential associations. The observed decrease in opioid misuse demonstrates a potential change in underlying risk factors with implications for overdose prevention efforts for youth and adolescents. As intentional misuse of opioids decreases, unintentional consumption of fentanyl may become a more common cause of opioid overdose for youth and adolescents. Therefore, awareness campaigns sharing information about fentanyl contamination of nonopioid substances (eg, ecstasy, cocaine), improving naloxone availability, and supporting drug checking efforts may be increasingly important tools for decreasing overdoses for younger populations. Future planned research using this pseudocohort approach will examine polysubstance use and evaluate how substance use differs by other sociodemographic characteristics.

### Limitations

This study has some limitations. Data were available on the NSDUH public-use files for single ages from 12 through 21 years. Beyond 21 years of age, the data were coded into larger age groups, and we were not able to continue our pseudocohort ages past that point.

Relevant NSDUH questions on opioid use were redesigned in 2015, and therefore data are not comparable before and after. The Substance Abuse and Mental Health Services Administration encourages caution when comparing prescription drug use data before 2015 and 2015 and later. Because this shift in data collection procedures affects all pseudocohorts that cross 2014 to 2015, we document this limitation but continue to compare patterns between pseudocohorts.

Estimates of opioid misuse will be underestimated as individuals who are institutionalized (ie, incarcerated, living within a group home) or homeless are less likely to be included within the survey and experience higher rates of opioid misuse. This may have affected our estimates, especially for older adolescents, because this population is more likely to experience homelessness or be incarcerated. However, this should not impact comparisons across the constructed pseudocohorts or reported trends, as the structure and design of the NSDUH did not change in this regard, and this underestimation should apply to the pseudocohorts nondifferentially.

An additional limitation is related to the increased availability of naloxone for overdose reversal in later cohorts. It is possible that individuals with opioid misuse were more likely to die following overdose in earlier years compared with later years when naloxone was more available. The potential for survivor bias, therefore, impacts the pseudocohorts differentially. However, we would have expected the rates of opioid misuse to increase in later years (because of more individuals surviving overdose and returning to use) rather than decrease as overdose reversal became more common. Therefore, any impact of survivor bias likely biased reported trends toward the null.

We did not combine 2020 NSDUH data with the earlier data (as suggested by NSDUH administrators) due to changes to the data in response to COVID-19. Drug overdose deaths increased, and many factors associated with vulnerability to substance misuse (eg, mental health, joblessness, homelessness) worsened during the COVID-19 pandemic. Trends described within this analysis do not account for changes in trends related to the pandemic and thus may introduce underestimation biases.^22,23^

Although there is evidence of underreporting of illegal behavior in the NSDUH, this bias is likely to be consistent across the years and should not impact the cohort analysis. Not all past-year opioid misuse reports were for opioids; some were prescription pain relievers based on write-in data that were not considered to be opioids. However, at least 96% of the reports of past-year opioid use were confirmed as opioids. During the years of data analyzed in this study, the NSDUH did not specifically ask about the use of illegally made fentanyl, but this is unlikely to impact our study’s finding because reported injection drug use and heroin use was uncommon.

## Conclusions

Estimating and plotting past-year opioid misuse by age and across pseudocohorts illuminates an important takeaway: youths and young adults of high school age have consistently misused fewer opioids in later pseudocohorts. The same improvements in opioid misuse have been seen for male and female individuals separately, and differences in opioid rates by sex have also diminished in later pseudocohorts. A decrease in drug availability and general exposure to the harms of opioid use could be contributing to this important finding. Examination of polysubstance use and how substance use differs by other sociodemographic characteristics should use this pseudocohort approach.

## Notes

Supplement 1. **eTable 1.** Past-Year Opioid Misuse Percentages and 95% CIs, by Age and Cohort Among Male Participants **eTable 2.** Past-Year Opioid Misuse Percentages and 95% CIs, by Age and Cohort Among Female Participants **eTable 3.** Comparison of Past-Year Opioid Misuse Percentages Between Sexes, by Age and Cohort **eTable 4.** Question Wording in the 2019 NSDUH Survey

Supplement 2.
Data Sharing Statement
