# Supplementary material for: Trends in Opioid Misuse Among Individuals Aged 12 to 21 Years in the US
Source: JAMA Netw Open. 2023 Jun 1;6(6):e2316276. doi: 10.1001/jamanetworkopen.2023.16276 (PMC10236235; doi:10.1001/jamanetworkopen.2023.16276)
Supplement: Supplement 1. — eTable 1. Past-Year Opioid Misuse Percentages and 95% CIs, by Age and Cohort Among Male Participants eTable 2. Past-Year Opioid Misuse Percentages and 95% CIs, by Age and Cohort Among Female Participants eTable 3. Comparison of Past-Year Opioid Misuse Percentages Between Sexes, by Age and Cohort eTable 4. Question Wording in the 2019 NSDUH Survey [file jamanetwopen-e2316276-s001.pdf]

Supplementary Online Content

Warren LK, Adams J, Bobashev G. Trends in opioid misuse among individuals aged 12 to 21 years in the US. *JAMA Netw Open*. 2023;6(6):e2316276. doi:10.1001/jamanetworkopen.2023.16276

eTable 1. Past-Year Opioid Misuse Percentages and 95% CIs, by Age and Cohort Among Male Participants

eTable 2. Past-Year Opioid Misuse Percentages and 95% CIs, by Age and Cohort Among Female Participants

eTable 3. Comparison of Past-Year Opioid Misuse Percentages Between Sexes, by Age and Cohort

eTable 4. Question Wording in the 2019 NSDUH Survey

This supplementary material has been provided by the authors to give readers additional information about their work.

© 2023 Warren LK et al. *JAMA Network Open*.

eTable 1. Past-Year Opioid Misuse Percentages and 95% CIs, by Age and Cohort Among Male Participants

AGE	2002 Cohort % (95% CI)	2005 Cohort % (95% CI)	2008 Cohort % (95% CI)	2011 Cohort % (95% CI)	2014 Cohort % (95% CI)
12	1.81 (1.12, 2.50)	2.26 (1.45, 3.07)	2.29 (1.18, 3.39)	2.26 (1.33, 3.18)	2.11 (1.16, 3.06)
13	3.94 (2.74, 5.14)	2.80 (1.81, 3.78)	2.73 (1.58, 3.87)	3.57 (2.35, 4.78)	2.16 (1.27, 3.05)
14	4.95 (3.55, 6.34)	4.65 (3.21, 6.08)	3.55 (2.23, 4.88)	4.11 (2.61, 5.62)	2.30 (1.01, 3.60)
15	5.07 (3.84, 6.29)	5.66 (4.34, 6.98)	6.91 (5.36, 8.47)	4.37 (2.74, 6.01)	2.68 (1.26, 4.10)
16	10.22 (8.52, 11.92)	8.78 (7.07, 10.49)	8.00 (5.74, 10.25)	5.33 (3.60, 7.07)	3.22 (1.87, 4.57)
17	10.50 (8.92, 12.09)	10.10 (8.13, 12.08)	8.52 (7.01, 10.04)	7.61 (5.66, 9.55)	4.01 (2.44, 5.58)
18	11.80 (9.59, 14.02)	10.03 (7.90, 12.16)	7.41 (5.04, 9.78)	4.49 (2.65, 6.34)	
19	13.37 (11.09, 15.65)	10.03 (7.89, 12.17)	9.25 (6.77, 11.73)	5.55 (4.00, 7.10)	
20	13.22 (10.56, 15.88)	12.64 (9.89, 15.39)	8.11 (5.46, 10.75)	5.01 (3.21, 6.82)	
21	10.12 (7.40, 12.84)	11.15 (8.09, 14.21)	7.70 (5.30, 10.10)		

% = Percentage; CI = Confidence Interval.

2002-2019 NSDUH Public-Use Files

© 2023 Warren LK et al. *JAMA Network Open*.

eTable 2. Past-Year Opioid Misuse Percentages and 95% CIs, by Age and Cohort Among Female Participants

AGE	2002 Cohort % (95% CI)	2005 Cohort % (95% CI)	2008 Cohort % (95% CI)	2011 Cohort % (95% CI)	2014 Cohort % (95% CI)
12	2.57 (1.55, 3.60)	3.26 (1.95, 4.58)	3.06 (1.89, 4.22)	3.34 (1.97, 4.72)	2.02 (1.11, 2.92)
13	4.60 (3.21, 5.99)	4.20 (2.81, 5.60)	3.89 (2.49, 5.28)	2.50 (1.54, 3.45)	2.10 (0.92, 3.28)
14	8.58 (6.51, 10.65)	5.54 (4.03, 7.06)	4.85 (3.43, 6.26)	4.83 (3.16, 6.50)	4.00 (2.37, 5.62)
15	8.79 (6.94, 10.64)	8.96 (7.21, 10.70)	8.32 (6.85, 9.79)	7.65 (5.57, 9.72)	5.02 (3.46, 6.59)
16	10.32 (8.67, 11.97)	11.17 (8.98, 13.36)	6.91 (5.13, 8.69)	6.90 (5.15, 8.65)	3.89 (2.41, 5.37)
17	11.28 (9.62, 12.95)	10.47 (8.35, 12.58)	6.26 (4.57, 7.95)	4.98 (3.25, 6.70)	4.00 (2.74, 5.26)
18	11.57 (9.33, 13.81)	9.21 (7.44, 10.99)	7.24 (5.02, 9.46)	6.38 (4.06, 8.70)	
19	11.03 (8.42, 13.65)	8.67 (6.13, 11.20)	7.87 (5.52, 10.22)	4.34 (2.71, 5.96)	
20	9.11 (6.70, 11.51)	8.49 (6.69, 10.29)	6.25 (4.60, 7.90)	5.61 (3.27, 7.95)	
21	7.63 (5.49, 9.77)	8.01 (5.56, 10.46)	7.59 (5.51, 9.68)		

% = Percentage; CI = Confidence Interval.

2002-2019 NSDUH Public-Use Files

© 2023 Warren LK et al. *JAMA Network Open*.

eTable 3. Comparison of Past-Year Opioid Misuse Percentages Between Sexes, by Age and Cohort

AGE	2002 Cohort		2005 Cohort		2008 Cohort		2011 Cohort		2014 Cohort	
	**Difference in % (95% CI)**	***P*-Value**	**Difference in % (95% CI)**	***P*-Value**	**Difference in % (95% CI)**	***P*-Value**	**Difference in % (95% CI)**	***P*-Value**	**Difference in % (95% CI)**	***P*-Value**
12	-0.76 (-2.04, 0.52)	0.24	-1.00 (-2.49, 0.48)	0.18	-0.77 (-2.47, 0.93)	0.37	-1.09 (2.90, 0.72)	0.24	0.09 (-1.32, 1.50)	0.90
13	-0.66 (-2.47, 1.15)	0.47	-1.41 (-3.23, 0.42)	0.13	-1.16 (-3.22, 0.91)	0.23	1.07 (-0.43, 2.57)	0.16	0.06 (-1.59, 1.71)	0.94
14	-3.63 (-6.19, -1.08)	0.005*	-0.89 (-3.08, 1.29)	0.42	-1.29 (-3.28, 0.69)	0.20	-0.72 (-3.06, 1.62)	0.55	-1.69 (-3.67, 0.29)	0.09
15	-3.73 (-5.83, -1.62)	0.001*	-3.30 (-5.36, -1.23)	0.002*	-1.41 (-3.37, 0.55)	0.16	-3.27 (-5.66, -0.88)	0.007*	-2.34 (-4.73, 0.04)	0.05
16	-0.10 (-2.52, 2.32)	0.94	-2.39 (-5.55, 0.77)	0.14	1.09 (-1.63, 3.8)	0.43	-1.57 (-4.26, 1.13)	0.25	-0.67 (-2.9, 1.56)	0.55
17	-0.78 (-2.85, 1.29)	0.46	-0.36 (-3.38, 2.65)	0.81	2.27 (-0.06, 4.59)	0.05	2.63 (0.12, 5.15)	0.04*	0.01 (-2.14, 2.15)	1.0
18	0.24 (-2.62, 3.09)	0.87	0.82 (-2.16, 3.80)	0.59	0.17 (-2.68, 3.03)	0.91	-1.89 (-4.67, 0.90)	0.18		
19	2.34 (-0.85, 5.52)	0.15	1.36 (-1.98, 4.71)	0.42	1.38 (-2.43, 5.19)	0.47	1.21 (-0.53, 2.96)	0.17		
20	4.11 (1.04, 7.19)	0.008*	4.15 (0.77, 7.53)	0.02*	1.86 (-1.23, 4.95)	0.24	-0.60 (-3.23, 2.04)	0.66		
21	2.49 (-0.69, 5.68)	0.12	3.14 (-0.63, 6.91)	0.10	0.11 (-2.44, 2.65)	0.93				

% = Percentage; CI = Confidence Interval; * Statistically significant at the 0.05 alpha level.
Note: These *P*-values have not been adjusted for multiple comparisons.
Note: Differences correspond to Male % - Female %.
2002-2019 NSDUH Public-Use Files

© 2023 Warren LK et al. *JAMA Network Open*.

eTable 4. Question Wording in the 2019 NSDUH Survey

Past-Year Pain Reliever Misuse	
**PRINTROYR1**	[IF PR12MON = 1] Earlier you reported having used certain **prescription pain relievers** during the past year. Now please think about whether you used any of these pain relievers in any way **a doctor did not direct you to use them.**<br>When you answer these questions, please think only about your use of the drug in any way **a doctor did not direct you to use it,** including:<br>▪ Using it without a prescription of your own<br>▪ Using it in greater amounts, more often, or longer than you were told to take it<br>▪ Using it in **any other way** a doctor did not direct you to use it
**PRY01**	[IF PR01=1] In the past 12 months, did you use [Pain Reliever Type] in any way **a doctor did not direct you to use it**?<br><br>DISPLAY IMAGE FOR [DRUG]<br><br>1 Yes<br>2 No<br>DK/REF
Past-Year Heroin Use	
**HELAST3**	[IF HE01 = 1 OR HEREF = 1] How long has it been since you **last** used heroin?<br><br>1 Within the past 30 days -- that is, since **[DATEFILL]**<br>2 More than 30 days ago but within the past 12 months<br>3 More than 12 months ago<br>DK/REF<br>PROGRAMMER: SHOW 12 MONTH CALENDAR
Age at First Use	
**PRY01a**	[IF PRFIRSTFLAG=1] Please think about the **first** time you **ever** used [Pain Reliever Type] in a way a doctor did not direct you to use it.<br><br>[IF PRY01=1] How old were you when you first used [Pain Reliever Type] in a way **a doctor did not direct you to use it**?<br><br>AGE: [(RANGE: 1 - 110)]<br>DK/REF
**HE02**	[IF HE01 = 1 OR HEREF = 1] How old were you the **first time** you used heroin?<br><br>AGE: [RANGE: 1 - 110]<br>DK/REF
Lifetime Substance Use Treatment	
**TX01**	[IF AL01 = 1 OR ALREF = 1 OR MJ01 = 1 OR MJREF = 1 OR CC01 = 1 OR

© 2023 Warren LK et al. *JAMA Network Open*.

	CCREF = 1 OR CK01 = 1 OR CKREF = 1 OR HE01 = 1 OR HEREF = 1 OR LS01a = 1 LSREF1 = 1 OR LS01b = 1 OR LSREF2 = 1 OR LS01c = 1 OR LS01d = 1 OR LS01e = 1 OR LS01f = 1 OR LSREF3 = 1 OR LS01h = 1 OR LS01i=1 OR LS01j=1 OR LS01k=1 OR IN01a =1 OR IN01b =1 OR IN01c = 1 OR IN01d = 1 OR IN01e = 1OR IN01f = 1 OR IN01g = 1 OR IN01h = 1 OR IN01h1=1 OR IN01i = 1 OR IN01ii=1 OR IN01j = 1 OR IN01l = 1 OR INREF = 1 OR ME01=1 OR MEREF=1 OR PRMISUSE12=1 OR PRL01=1 OR PRL02=1 OR TRMISUSE12=1 OR TRL01=1 OR TRL02=1 OR STMISUSE12=1 OR STL01=1 OR STL02=1 OR SVMISUSE12=1 OR SVL01=1 OR SVL02=1] Have you **ever received** treatment or counseling for your use of alcohol or any drug, not counting cigarettes?
Exposure to Prevention Messages	
**YE24a**	[IF YE09 = 1 OR YE09a = 1] **During the past 12 months** have you had a special class about drugs or alcohol in school?
**YE24b**	[IF YE09 = 1 OR YE09a = 1] **During the past 12 months** have you had films, lectures, discussions, or printed information about drugs or alcohol **in** one of your regular school classes such as health or physical education?
**YE24c**	[IF YE09 = 1 OR YE09a = 1] **During the past 12 months** have you had films, lectures, discussions, or printed information about drugs or alcohol **outside of** one of your regular classes such as in a special assembly?
**YE25**	[IF YE09 = 1 OR YE09a = 1] **During the past 12 months** have you seen or heard any alcohol or drug prevention messages from sources outside school such as posters, pamphlets, radio, or TV?<br><br>[IF YE09 = 2 OR DK/REF AND YE09a = 2 OR DK/REF] **During the past 12 months** have you seen or heard any alcohol or drug prevention messages from sources such as posters, pamphlets, radio, or TV?
Questions for Perceived Availability of Heroin	
**RK02e**	How difficult or easy would it be for you to get some **heroin**, if you wanted some?<br><br>1 Probably impossible<br>2 Very difficult<br>3 Fairly difficult<br>4 Fairly easy<br>5 Very easy<br>DK/REF

© 2023 Warren LK et al. *JAMA Network Open*.
