# Supplementary material for: Trends in Opioid Misuse Among Individuals Aged 12 to 21 Years in the US
Source: JAMA Netw Open. 2023 Jun 1;6(6):e2316276. doi: 10.1001/jamanetworkopen.2023.16276 (PMC10236235; doi:10.1001/jamanetworkopen.2023.16276)
Supplement: Supplement 2. — Data Sharing Statement [file jamanetwopen-e2316276-s002.pdf]

Data Sharing Statement

Warren. Trends in Opioid Misuse Among Individuals Aged 12 to 21 Years in the US. *JAMA Netw Open*. Published June 01, 2023. doi:10.1001/jamanetworkopen.2023.16276

Data
Data available: Yes
Data types: Deidentified participant data
How to access data: https://www.datafiles.samhsa.gov/dataset/national-survey-drug-use-and-health-2019-nsduh-2019-ds0001
When available: With publication

Supporting Documents
Document types: None

Additional Information
Who can access the data: General public.
Types of analyses: For any purpose.
Mechanisms of data availability: Open access.
Any additional restrictions: N/A
